# PARP activation promotes nuclear AID accumulation in lymphoma cells

**DOI:** 10.18632/oncotarget.7603

**Published:** 2016-02-23

**Authors:** Sandra Tepper, Julia Jeschke, Katrin Böttcher, Angelika Schmidt, Kathrin Davari, Peter Müller, Elisabeth Kremmer, Peter Hemmerich, Ines Pfeil, Berit Jungnickel

**Affiliations:** ^1^ Department of Cell Biology, Institute of Biochemistry and Biophysics, Center for Molecular Biomedicine, Friedrich-Schiller University, 07745 Jena, Germany; ^2^ Institute of Clinical and Molecular Biology, Helmholtz Center Munich, 81377 Munich, Germany; ^3^ Institute of Molecular Immunology, Helmholtz Center Munich, 81377 Munich, Germany; ^4^ Imaging Facility, Leibniz- Institute on Aging – Fritz Lipmann Institute, 07745 Jena, Germany

**Keywords:** activation-induced cytidine deaminase, DNA damage, protein stability, poly (ADP-ribose) polymerase, lymphoma

## Abstract

Activation-induced cytidine deaminase (AID) initiates immunoglobulin diversification in germinal center B cells by targeted introduction of DNA damage. As aberrant nuclear AID action contributes to the generation of B cell lymphoma, the protein's activity is tightly regulated, e.g. by nuclear/cytoplasmic shuttling and nuclear degradation. In the present study, we asked whether DNA damage may affect regulation of the AID protein. We show that exogenous DNA damage that mainly activates base excision repair leads to prevention of proteasomal degradation of AID and hence its nuclear accumulation. Inhibitor as well as knockout studies indicate that activation of poly (ADP-ribose) polymerase (PARP) by DNA damaging agents promotes both phenomena. These findings suggest that PARP inhibitors influence DNA damage dependent AID regulation, with interesting implications for the regulation of AID function and chemotherapy of lymphoma.

## INTRODUCTION

Secondary immunoglobulin diversification by somatic hypermutation and class switch recombination in germinal centers is the major prerequisite for maturation of the humoral adaptive immune response [1, 2]. However, it also includes the danger of genetic instability, as other cellular genes may also be affected by mutagenesis or chromosomal translocations [3, 4]. Accordingly, both the enzymes introducing DNA damage as well as the machinery responsible for DNA repair need to be tightly regulated in germinal center B cells to on the one hand promote their efficient function, but on the other hand prevent their deregulation leading to cellular transformation and hence lymphomagenesis.

AID is the key enzyme initiating hypermutation and class switch recombination by transcription-coupled deamination of cytidines [5–7]. Expression and activity of the AID protein is regulated at multiple levels, including its cellular localization, posttranslational modifications and stability [8–10]. While the main compartment of AID activity is clearly the nucleus, most of the protein is retained in the cytoplasm in the steady state situation based on the coordinate action of heat shock proteins and eEF1A [11–14]. A constant nuclear/cytoplasmic shuttling of AID is mediated by a N-terminal structural nuclear localization sequence (NLS) and a nuclear export sequence (NES) encompassing the C-terminal 14 amino acids [15–18]. Trapping of AID in the nucleus, e.g. by inhibition of CRM1/exportin1 via leptomycin B, leads to its rapid degradation, which involves the proteasome and may occur in both a ubiquitination-dependent as well as -independent manner [9, 19].

AID can also be modified by phosphorylation at serine (S38) and threonine (T27, T140) residues [20–22]. S38 phosphorylation by protein kinase A (PKA) in the nucleus has been shown to be involved in binding of AID to replication protein A (RPA), a process that increases AID processivity on DNA and hence the efficiency of AID-mediated mutagenesis [23]. While AID may be detected at many gene loci [24], PKA activity appears to be specifically targeted to immunoglobulin loci in germinal center B cells [25]. Locus-specificity of somatic hypermutation is also increased by differential DNA repair of AID-induced DNA lesions, which is error-prone in immunoglobulin genes but error-free in many other cellular genes that are not affected by hypermutation in the germinal center [26].

While proficient AID activity is required at hypermutating gene loci in B cells to ensure rapid adaptation of the humoral immune response, a major risk for genetic instability caused by AID activity can occur upon introduction of excessive damage into the genome. This accumulation of DNA lesions may overwhelm the DNA repair capacity of the cell or give rise to multiple strand breaks that can be processed to chromosomal translocations [27, 28]. Such a scenario could be prevented by negative feedback loops by which DNA damage-associated signals avoid introduction of more damage by counteracting nuclear AID activity. However, at least one positive feedback loop has also been identified: introduction of strand breaks promotes AID phosphorylation and hence its activity at the immunoglobulin locus [29, 30].

In the present study, we asked whether DNA damage affects other aspects of posttranslational regulation of AID, in particular its nuclear localization and/or degradation. Unexpectedly, we found that cytotoxic drugs that activate base excision repair (which is also activated by AID) lead to the nuclear accumulation and stabilization of the AID protein. Studies using inhibitors as well as knockout cell lines indicate that activation of poly(ADP-ribose) polymerase (PARP) is required for efficient nuclear AID accumulation and stabilization. These findings define the first molecular pathway that may lead to nuclear accumulation of AID, with interesting potential implications for the regulation of AID function as well as for lymphoma therapy.

## RESULTS

### Proteasomal nuclear degradation of AID-GFP fusions in human and chicken B cells

To perform our studies on AID localization and degradation in easily tractable hypermutating B cell lines, we first tested whether the degradation of AID in human Raji and chicken DT40 B cell lymphoma lines follows the same mechanism observed before for human BL2 cells [9]. We used human AID fusions carrying a GFP gene either at the C-terminus or N-terminus and containing or lacking the last 14 amino acids comprising the AID NES. In the steady state situation, the wild-type AID fusion proteins were localized in the cytoplasm, while the truncated proteins accumulated in the nucleus (Supplementary Figure S1A). Also, clones with AID fusions containing a NES were on average substantially more highly fluorescent than those lacking it, irrespective of whether GFP was fused to the N- or C-terminus (Supplementary Figure S1B and S1C). Induction of degradation of AID fusions, measured by loss of fluorescence relative to the control sample, required the addition of the nuclear export inhibitor leptomycin B (LMB) or translational inhibition by cycloheximide (CHX) for the wild-type protein; the strongest effect was induced by the combination of these drugs (Supplementary Figure S1D). While the endogenous AID protein disappeared after 8 hours of CHX/LMB treatment, a GFP control protein remained stable (Supplementary Figure S1E). Obviously, the proteasome was involved in nuclear AID degradation, as its inhibition with MG 132 decreased AID degradation (Supplementary Figure S1D). Accordingly, in human Raji cells and chicken DT40 lymphoma cells, the AID protein is degraded by the ubiquitin-proteasome pathway in the nucleus, as shown before for BL2 cells [9]. For further experiments, AID fusions with GFP at the C-terminus were used.

### DNA damage increases AID protein stability in the nucleus

**Figure 1:**
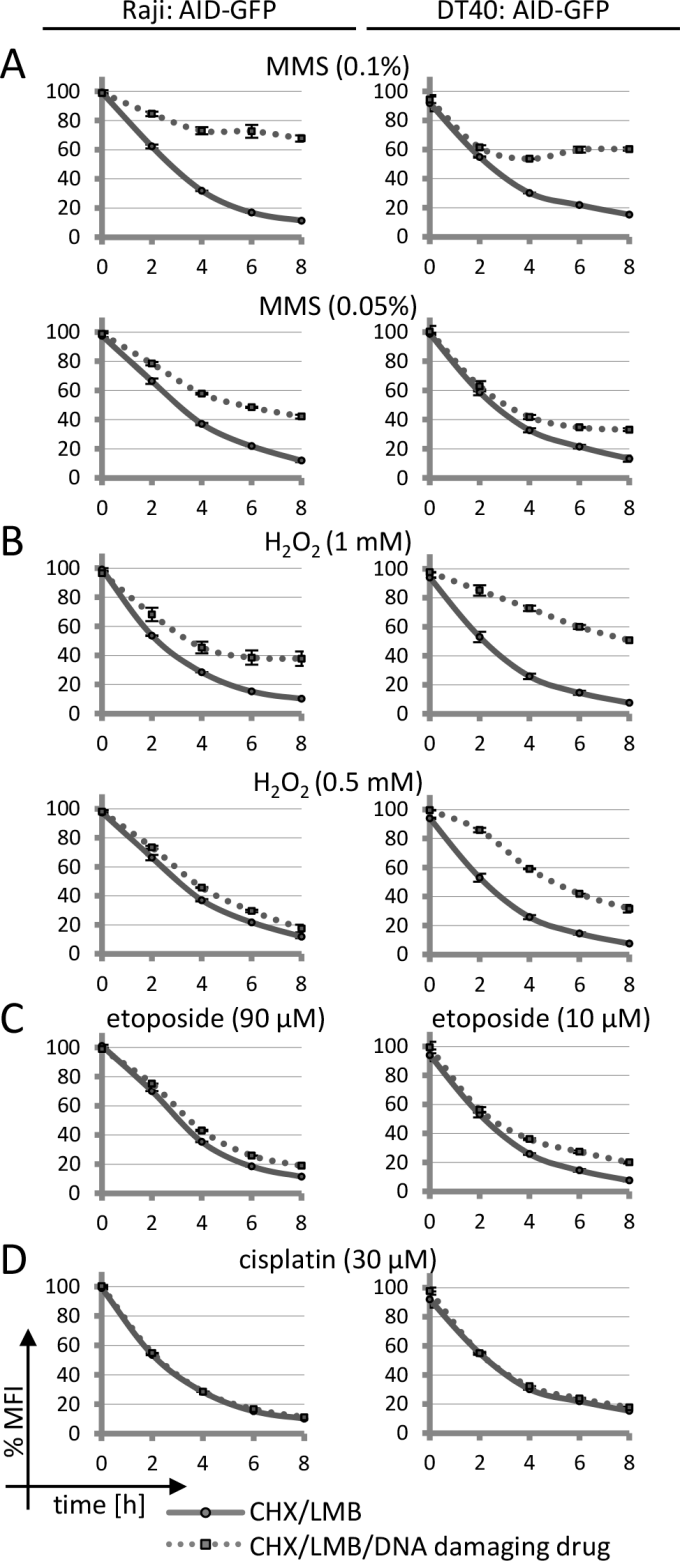
Effects of DNA damage on AID-GFP protein degradation **A–D.** FACS analysis of human Raji or chicken DT40 B cell lines stably transfected with an AID-GFP construct: degradation of the AID-GFP proteins trapped in the nucleus upon treatment with the indicated drugs. Untreated cells are set to 100% MFI (geometric mean fluorescence intensity). Relative MFI values are given as a function of time with standard deviation for duplicates. The experiment was performed with two independent transfectants and is representative of more than three independent experiments.

To analyze whether (AID-induced) DNA damage might impact on AID degradation, we applied cytotoxic drugs. Intriguingly, methyl methanesulfonate (MMS) and H_2_O_2_, both of which similarly to AID initially activate base excision repair, also decreased the degradation of AID fusions trapped in the nucleus (Figure 1A and 1B). Etoposide, a Topoisomerase II inhibitor inducing DNA double strand breaks, caused a very moderate inhibition of AID degradation in chicken lymphoma cells but barely so in human Raji lymphoma cells (Figure 1C). The nucleotide excision repair inducing drug cisplatin did not have this effect at any concentration tested (Figure 1D). All drugs tested caused DNA damage as indicated by Chk1 phosphorylation (Supplementary Figure S2) at the concentrations used. We also observed a restricted AID degradation under MMS and H_2_O_2_ in mouse CH12F3 lymphoma cells (Supplementary Figure S3 [31]).

**Figure 2:**
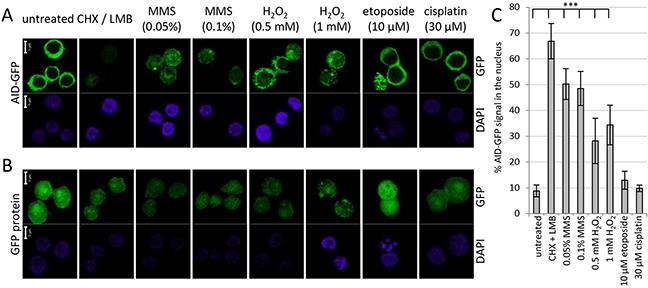
Effects of DNA damage on protein localization of AID-GFP Localization of AID-GFP **A.** and the GFP protein itself **B.** in DT40 lymphoma cells after 6 hours of treatment with the indicated drugs; scale bar: 5 μm. **C.** Quantification of the experiment shown in A, analyzing 7 to 12 cells for each condition. ***: *p* < 0.0001 (student's *t*-test).

Inhibition of proteasomal AID degradation by DNA damage might indicate that the protein can no longer enter the nucleus in order to be degraded. However, confocal microscopy revealed that in contrast to this assumption, MMS and H_2_O_2_ induced a substantial nuclear accumulation of AID-GFP fusions even if no LMB was added to the assay (Figure 2A and 2C). No effect was seen for the GFP protein itself (Figure 2B), implying that the effects are implemented via the AID portion of the fusions. Cisplatin and etoposide did not show significant effects on AID localization, even at etoposide concentrations that induced nuclear fragmentation in many cells. We conclude that drugs activating base excision repair lead to a nuclear accumulation of AID that is coupled to inhibition of nuclear proteasomal AID degradation. These findings imply that a processing intermediate of the damage induced by MMS and H_2_O_2_ leads to a reaction that interferes with nuclear AID degradation, and thus causes nuclear AID accumulation.

**Figure 3:**
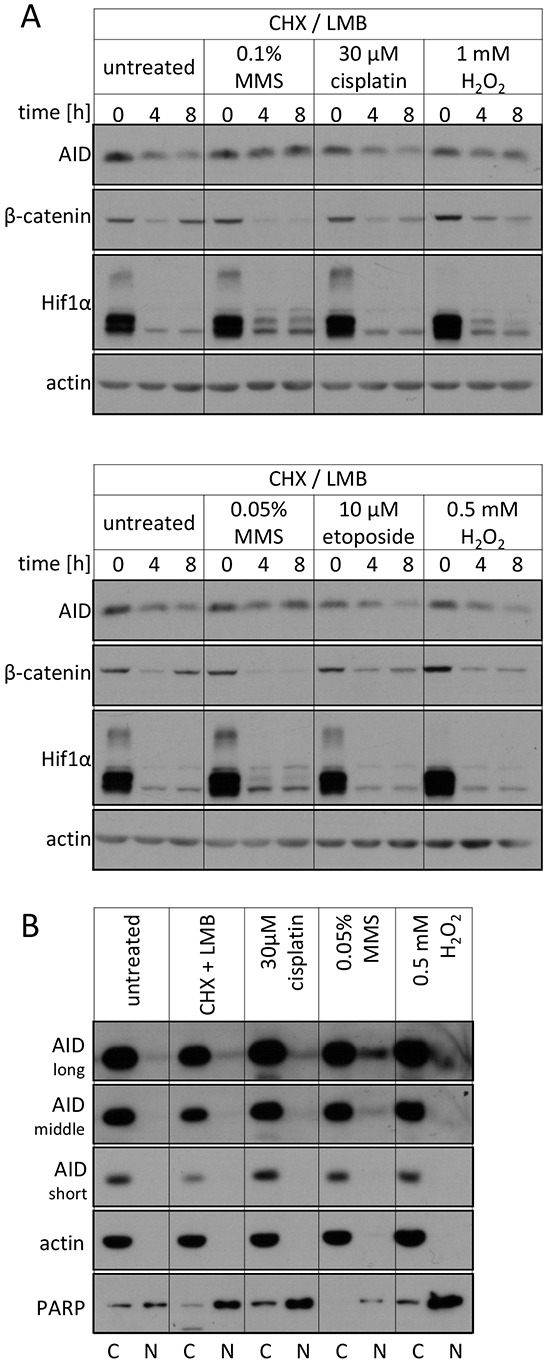
Effects of DNA damage on the endogenous AID protein **A.** Western blot analysis of total endogenous AID protein levels in Raji B cells upon treatment with the indicated drugs. Representative blots of 3 independent experiments are shown. β-catenin and Hif1α serve as controls for irrelevant unstable proteins. **B.** Western blot analysis of endogenous AID protein levels in cytoplasmic and nuclear fractions of Raji B cells upon treatment with the indicated substances for 5 hours. Three different exposures for AID are shown. The quality of separation was tested with the compartment markers actin (cytosolic) and PARP (nuclear). C: cytoplasmic, N: nuclear. Data are representative of two independent experiments.

To confirm that a stabilization of AID by DNA damage is also observed for the endogenous protein, we studied AID degradation by Western blots. MMS- and H_2_O_2_-derived DNA lesions also caused a stabilization of the endogenous AID protein (Figure 3A), while cisplatin and etoposide had no such effect. Also, fractionation experiments showed that the endogenous AID protein accumulates in the nucleus upon MMS- and H_2_O_2_, but not cisplatin treatment (Figure 3B). These results substantiate an altered subcellular localization and an increased protein stability of nuclear AID as a consequence of exogenous DNA damage inducing the base excision repair pathway.

### PARP inhibition prevents AID stabilization and nuclear accumulation

MMS and H_2_O_2_ both mainly activate the base excision repair pathway, as does AID itself. This repair pathway is based on the excision of modified bases by different DNA glycosylases chosen depending on the damaged base, followed by incision at the abasic site by apurinic endonucleases (APE1 and 2), and ligation involving re-insertion of the correct base. This last step involves recruitment of PARP to the strand breaks generated, thus triggering poly(ADP-ribosylation) of PARP itself and other proteins, and leading to recruitment of multiple factors for enhanced efficacy of DNA repair. Indeed, of the drugs used in this study, only MMS and H_2_O_2_ were able to efficiently induce PARylation of proteins (Supplementary Figure S2).

**Figure 4:**
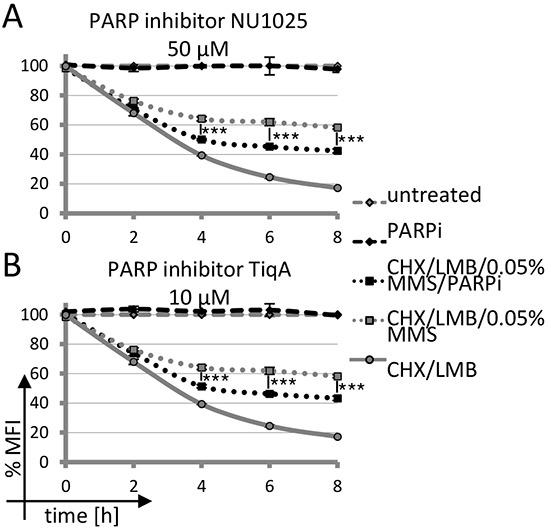
Impact of PARP inhibition on AID-GFP degradation upon DNA damage **A, B.** Kinetics of degradation of nuclear AID-GFP fusions in Raji B cells treated with 0.05% MMS and PARP inhibitor. Untreated cells are set to 100% MFI. Relative MFI values are given; significance analysis was performed for six technical replicates (student's *t*-test), ***: *p* < 0.001. Data are representative of three independent experiments.

In order to assess whether intermediates of base excision repair may lead to nuclear AID stabilization, we first analyzed whether inhibitors of PARP affect the process. Indeed, inhibition of PARP activity with several different chemical inhibitors substantially interfered with AID stabilization by MMS treatment in human, chicken and mouse cells (Figure 4A and 4B and Supplementary Figure S4), implying a role for PARP activation in nuclear AID accumulation. Thus, activation of PARP by MMS treatment appears to be responsible for efficient nuclear stabilization and accumulation of AID.

### Nuclear AID stabilization is impaired in PARP-1 knockout cells

**Figure 5:**
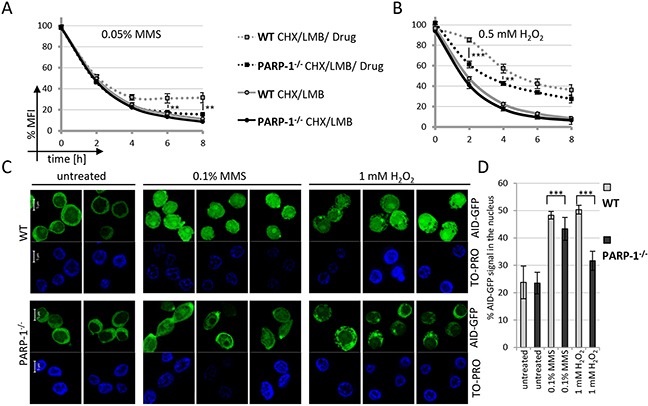
Nuclear AID stabilization is impaired in PARP-1 knockout cells FACS analysis of nuclear degradation of AID-GFP in wild-type and PARP-1^−/−^ cells and stabilization upon treatment with MMS **A.** and H_2_O_2_ **B.** Untreated cells are set to 100% MFI. Relative MFI values of five independent clones per condition are given as a function of time with the indicated standard deviation. *P*-values for wild-type compared to PARP-1^−/−^ cells after CHX / LMB / MMS treatment for different time points are given (student's *t*-test), **: *p* < 0.01, ***: *p* < 0.001. Data are representative of two independent experiments each. **C.** Subcellular localization of AID-GFP fusions 4 hours after treatment with MMS and H_2_O_2_; scale bar: 5 μm. Data are representative of two independent experiments. Note some focal accumulation of AID at a single spot in the cytoplasm observed in this and some other experiments. **D.** Quantification of the experiment shown in C, analyzing 15 cells each from two independent clones per condition. ***: *p* < 0.0001(student's *t*-test).

To rule out that an off-target activity of the PARP inhibitors caused the observed effect on AID stabilization, we wanted to confirm our results in a clearcut genetic system. In mammalian cells, however, PARP-1 and PARP-2 both contribute to DNA repair, making genetic analyses complicated. We thus resorted to using PARP-1 knockout DT40 B lymphoma cells, as these apparently do not harbor a PARP-2 gene [32]. The kinetics of degradation of AID-GFP fusions trapped in the nucleus by LMB was similar in wild-type and PARP-1^−/−^ DT40 cells (Figure 5A). However, additional MMS- or H_2_O_2_-treatment led to a significantly reduced AID stabilization in the PARP-1^−/−^ cells (Figure 5A and 5B) as compared to wild-type cells. In agreement with this, MMS- or H_2_O_2_-treatment led to a significantly lower nuclear AID accumulation in PARP-1^−/−^ cells (Figure 5C and 5D). We thus conclude that nuclear activation of PARP, induced here by DNA damage, is capable of promoting nuclear stabilization of the inherently unstable AID protein, leading to its accumulation at its site of action.

### Stabilization and nuclear accumulation of AID mutants

A previous study has shown a positive feedback loop of AID activation by phosphorylation, dependent on AID activity-induced DNA damage [30]. To assess whether AID stabilization in the nucleus depends on its activity or phosphorylation, we generated AID mutants defective in either process (Supplementary Figure S5A). Mutagenesis of the zinc coordinating residues H56 and E58 perturbs the cytidine deaminase active center of AID [33]. Enzymatic inactivation is also achieved by mutagenesis of R19 and R24 in a loop that extends close to the active center in wild-type AID but apparently restricts substrate access in the respective mutant [34]. Mutagenesis of S38 impairs PKA-mediated phosphorylation and thus RPA binding of AID, an effect that is exacerbated by additional mutagenesis of T27 [35].

The catalytic (but not the phosphorylation) mutants of the AID-GFP fusion proteins showed a clearly reduced protein expression (Supplementary Figure S5B). However, these differences in protein levels are apparently not due to major differences in protein degradation (Supplementary Figure S5C), as this occurred to a comparable rate in all mutants. Most importantly, though, all the mutant AID proteins were stabilized in the nucleus by MMS treatment, as was the wild-type protein (Supplementary Figure S5D in comparison to S5C). We thus conclude that in the experimental system used here, the AID activity exerted by the AID-GFP fusions alone is not sufficient to promote their stabilization to a degree measurable with our current assays. Also, mutants with defective catalytic function or impaired activation by PKA still accumulate in the nucleus upon exogenously applied DNA damage.

### Nuclear AID stabilization as a consequence of cancer therapy

As AID stabilization in the nucleus was mostly observed using cytotoxic drugs, we wished to assess the relevance of this finding to cancer therapy. MMS is a DNA alkylating drug [36], as are other chemotherapy agents commonly used in lymphoma therapy, such as cyclophosphamide [37]. Its active metabolite alkylates N7 of guanine and generates DNA crosslinks, which are resolved by base excision repair [38, 39]. Indeed, we found that 4-hydroperoxy-cyclophosphamide positively affects AID stabilization (Supplementary Figure S6A) as well as nuclear AID accumulation (Supplementary Figure S6B and S6C) in human Burkitt's lymphoma cells at a concentration at or above that found in cancer patients upon treatment [40, 41]. We conclude that AID may be stabilized in the nucleus during lymphoma therapy using alkylating drugs such as cyclophosphamide.

## DISCUSSION

In the present study, we show that PARP inhibition counteracts nuclear stabilization and accumulation of the AID protein upon treatment with alkylating drugs causing exogenous DNA damage. We thereby identify PARP(−1) as the first molecule that, upon induction of DNA damage, may increase the nuclear concentration of this enzyme critical for immunoglobulin diversification and lymphomagenesis. As nuclear AID is highly more active in immunoglobulin mutagenesis than its cytoplasmic variant (Supplementary Figure S7), our data suggest that this mechanism might contribute to increased mutagenesis during lymphoma treatment.

Two previous studies have observed nuclear AID accumulation in adherent (non-B) cells upon induction of DNA strand breaks [42] or treatment with etoposide [43]. In both cases, nuclear AID accumulation affected only a minor proportion of the cells, in contrast to the substantial and homogenous AID accumulation we have observed in all the B cell lines used in this study. Notably, the most potent inducers of nuclear AID accumulation in our study were drugs inducing DNA alkylation and subsequent base excision repair (which is also induced by AID) [36, 38], rather than drugs inducing DNA strand breaks. Also, in case of etoposide treatment, only a very moderate (and PARP-independent, data not shown) nuclear AID stabilization was observed in the chicken lymphoma cell line in association with nuclear fragmentation. These findings may imply different DNA damage responses and different mechanisms of nuclear AID accumulation in B versus non-B cells, and in fact, we did not observe nuclear AID accumulation upon MMS treatment in several adherent cell lines analyzed (data not shown).

For the B cells undergoing immunoglobulin diversification studied here, we show for the first time that nuclear AID accumulation is accompanied by interference with nuclear proteasomal AID degradation, allowing a first glance at the molecular mechanism(s) involved. Also, we identify PARP(−1) as one responsible enzyme for this phenomenon. Presently, the PARP family consists of 17 members and the ubiquitous nuclear PARP-1 [44] is essential for the repair of DNA single-strand breaks via the base excision repair pathway [45] and responsible for around 90% of ADP-ribose polymer synthesis after DNA damage. Together with PARP-2, homo- and heterodimers can be formed and a role for PARP-2 in base excision repair was also detected [46]. Currently, in human cells we cannot distinguish by which PARP enzyme the effects observed are mediated, as the inhibitors used in our studies should target both PARPs. However, PARP-1 is the more likely candidate as deduced from its prominent activity.

It also remains to be seen whether AID stabilization upon PARP activation involves direct or indirect interaction of AID with (activated) PARP, modulation (e.g. poly(ADP-ribosylation)) of a protein involved in proteasomal AID degradation, or e.g. PARP-mediated activation of another DNA processing or DNA damage signaling pathway affecting nuclear AID stability. DNA damage is signaled to the cell by a variety of pathways, including e.g. checkpoint signaling, activation of MAP kinases or induction of NFκB activity [47–49]. In stark contrast to these mostly global DNA damage signals, PARP activation delivers a local signal, and thus triggers highly localized effects [50]. PARP accumulation at sites of DNA damage or at single strand breaks leads to automodification of PARP by long and branched ADP-ribose polymers. These serve as binding sites for DNA repair factors as well as other proteins, thereby enhancing the efficacy of DNA damage processing and repair [50]. Therefore, PARP-1/2, are powerful targets to increase the efficiency of cytotoxic chemotherapeutic agents inducing DNA damage, and PARP inhibitors are thus frequently used in combination therapy with alkylating and other cytotoxic agents.

We presume that in a physiological situation, PARP triggers AID accumulation directly at the site of DNA damage, as its PARylation function is required for the effect. In order to observe a global accumulation of AID in the nucleus of B cells upon PARP activation, we needed to induce substantial global DNA damage using high doses of exogenously applied DNA damaging agents, comparable to a patient's situation during chemotherapy, but potentially precluding the detection of foci that were apparent in a previous study [42]. Even though AID can be found at many gene loci in B cells undergoing immunoglobulin diversification, only some of these show evidence of DNA damage induction [24, 51]. It is thus not surprising that under steady state conditions in the absence of exogenously induced DNA damage, we cannot detect an influence of PARP on AID degradation. Given the higher AID activity in primary cells or *in vivo*, it will be worthwhile to study PARP effects on overall nuclear AID stability in other experimental systems, although our knowledge of PARP biology makes strong global effects unlikely.

Local effects of PARP, however, may be highly interesting in the context of immunoglobulin diversification, which requires locus-specific regulation of AID activity as well as of DNA repair processes. PARP is not only activated upon DNA damage to promote DNA repair, but also interacts with several transcription factors and affects transcriptional activity of gene loci [52, 53], among them the hypermutating Bcl-6 locus [54]. In addition, PARP was reported to affect DNA repair fidelity and pathway choice during immunoglobulin diversification [32, 55–60]. It will thus be highly interesting to study whether PARP affects AID stability and activity in a locus-specific manner in B cells undergoing immunoglobulin diversification, and if so, how activation of PARP is regulated in immunoglobulin genes as compared to other genes not undergoing diversification.

While an understanding of a potential physiological AID regulation via PARP will likely require more sophisticated experimental approaches, the form of AID regulation observed here may be of special importance in case of chemotherapy. According to our findings, such treatments may enhance nuclear AID accumulation, with potential impact on AID-induced mutagenesis and tumorigenesis once AID expression becomes deregulated. AID is a potent mutator that can induce aberrant somatic hypermutation of non-immunoglobulin genes and even genome kataegis [61–65]. Simultaneous application of PARP inhibitors appears to reduce nuclear AID accumulation and might thus preserve cells from aberrant AID function. Combined chemotherapy with additional PARP inhibitors is the subject of recent investigations and clinical trials [66, 67], e.g. for cyclophosphamide [68]. Our study shows that PARP(−1) inhibition or inactivation reduces the DNA damage-induced stabilization and accumulation of nuclear AID, providing an additional rationale for this therapeutic approach in lymphoma therapy.

## MATERIALS AND METHODS

### Cell culture and transfection

The human Burkitt's lymphoma cell line Raji and the chicken lymphoma cell lines DT40 Cre1 and DT40 ψV^−^ were cultured as described before [69]. The mouse CH12F3 B cells were maintained in RPMI 1640 supplemented with 10% FCS, 0.05 mM 2-mercaptoethanol (Sigma) and 10 mM Hepes (Invitrogen). The DT40 PARP-1 knockout cell set was provided by S. Takeda [32]. Transfections were carried out as described before [69] with a Gene Pulser Xcell (Bio Rad) set at 50 μF and 800 V for DT40 cells, at 850 μF and 250 V for Raji cells and at 400 μF and 400 V for CH12F3 cells. Stable transfectants were selected by addition of 0.5 μg/ml puromycin (Sigma Aldrich) and further tested by flow cytometry and Western blot. For analysis of somatic hypermutation, DT40ψV^−^ AID^−/−^ cells were stably transfected with the respective vectors, the resultant single cell clones were directly cultured for 19 days and stained with anti-chicken-IgM-PE (8310-09, Southern Biotech) before flow cytometry analysis.

### Plasmids and site-directed mutagenesis

pCAGGs vectors containing AID-GFP fusions cloned at the EcoRI site, bringing the AID gene under the transcriptional control of the chicken beta-actin promoter [70], were obtained from J. Bachl. For site-directed mutagenesis, the human AID coding sequence was introduced into the EcoRI site of pBluescriptKS (Stratagene). PCR was performed for 30 cycles with primers annealing at 60°C for 7 min and elongation at 72°C for 1 min, using the primers depicted in Supplementary Table S1. Subsequently, the non-mutated strand was cut with DpnI (Fermentas) and the mutated DNA was introduced into *E. coli* DH5α. The T27A/S38A double mutant was created by the introduction of the T27A mutation into the S38A mutant, while the R19E/R24E and H56R/E58Q double mutants were generated in a single mutagenesis step. Appropriate AID clones were confirmed by sequence analysis and subcloned into the pCAGGs vector.

### Induction of DNA damage and analysis of AID localization and degradation

DNA damage was induced by the following agents: etoposide (10 - 90 μM, Sigma Aldrich), cisplatin (30 μM, Ribosepharm), methyl methanesulfonate (MMS, 0.05 - 0.1%, Merck), and H_2_O_2_ (0.5 - 1 mM, Sigma-Aldrich). 4-hydroperoxy-cyclophosphamide was purchased from NIOMECH-IIT GmbH in aliquots, and for each experiment a fresh aliquot was dissolved in water and used directly. Protein translation was inhibited by addition of cycloheximide (CHX, 20 μg/ml, Sigma-Aldrich) and AID nuclear export was abrogated with leptomycin B (LMB, 5 ng/ml, Sigma-Aldrich). For additional treatment with inhibitors, the following final concentrations were used: MG132 (Calbiochem^®^): 10 μM; TiqA (Sigma-Aldrich): 10 μM; NU1025 (Santa Cruz): 50 μM and 3-Aminobenzamide (3-AB, Calbiochem^®^): 1 mM. For degradation kinetics, cells were analyzed using a CantoII (Becton Dickinson) in two hour intervals for a period of 8 hours followed by data assessment using FlowJo Software. GFP signals of living cells (identified by forward scatter analysis) were calculated as relative MFI (geometric mean fluorescence intensity) percentages, setting the MFI of untreated cells to 100 percent.

For confocal microscopy, cells were treated with the indicated agents for 4 to 6 hours. A total of 5×10^5^ cells in 1 ml were transferred onto cover slips precoated with poly-L-lysine (Sigma-Aldrich) and incubated for 15 min at 37°C, followed by 15 min fixation with 2% paraformaldehyde (Carl Roth) at room temperature. After washing with PBS, cells were permeabilized by 15 min treatment with 0.15% Triton-X-100 in PBS (Sigma-Aldrich) and subsequently stained with 100 ng/ml DAPI (Invitrogen) or 1 μM TO-PRO-3 (ThermoFisher scientific). Samples were scanned with a Zeiss LSM 510 laser scanning confocal device using a 63x Plan-Apochromat oil objective (Carl Zeiss). GFP and DAPI or TO-PRPO-3 were excited by laser light of 488 nm and 405 nm or 633 nm wavelength, respectively. Each signal was scanned independently by the multitracking function of the LSM 510 unit. Within each experiment, the 488 nm laser light was used at constant intensity in order to visualize changes in GFP intensities.

Quantification of confocal data was performed with the ZENblue software of Carl Zeiss Jena. For each cell, 6 ROIs (region of interest) were defined for the nucleus and 6 ROIs for the cytoplasm. The GFP MFI was determined by the software and the arithmetic average of the GFP signal in the nucleus plus the cytoplasm was set to 100%.

### Expression analysis of AID and AID mutants

12 to 13 days after electroporation of 1×10^7^ cells, single cell clones were analyzed for GFP signals by FACS analysis. FACS data were analyzed using FlowJo Software (Tree Star Inc., USA), gating for viable cells through scatter analyses. Western blots were done as described previously [71], using the following antibodies: α-AID (clone EK2/5G9, E.K.), α-GFP (B-2, Santa Cruz Biotechnology Inc., USA), α-actin (A-2066, Sigma-Aldrich), α-tubulin (ab59680, Abcam), α-PARP (ab32071, Abcam), α-P-Chk1 (Ser345) (Cell Signalling Technologies #2341), α-PARylation (ALX-202-043, Enzo), α-β-catenin (610154, BD transduction laboratories) and α-Hif1α (Cell Signalling Technologies #3716).

For cell fractionation, 1×10^6^ cells were resuspended in 1 volume of buffer NE-A (10 mM HEPES, pH 7.6; 30 mM KCl; 2 mM MgCl_2_; 0.1 mM EDTA;1 mM DTT; supplemented with proteinase inhibitor (Roche)) for 10 min on ice. After addition of another volume of buffer NE-A including 0.2% NP40 and 5 min incubation on ice, nuclei were separated by centrifugation (5 min, 700 rfc, 4°C). The supernatant is the cytoplasmic fraction. Nuclei were washed in PBS, resuspended in buffer NE-B (20 mM HEPES, pH 7.9; 420 mM NaCl; 25% glycerol; 0.2 mM EDTA; 1 mM DTT supplemented with proteinase inhibitor) and incubated on ice for 10 min. Following 3 freeze thaw cycles, the sample was further centrifuged (10 min, 15,000 rpm, 4°C) and the soluble fraction was collected.

## SUPPLEMENTARY FIGURES AND TABLE


