# Supplementary material for: PARP activation promotes nuclear AID accumulation in lymphoma cells
Source: Oncotarget. 2016 Feb 23;7(11):13197–208. doi: 10.18632/oncotarget.7603 (PMC4914351; doi:10.18632/oncotarget.7603)
Supplement: Supplementary file 1 [file oncotarget-07-13197-s001.pdf]

# SUPPLEMENTARY FIGURES AND TABLE

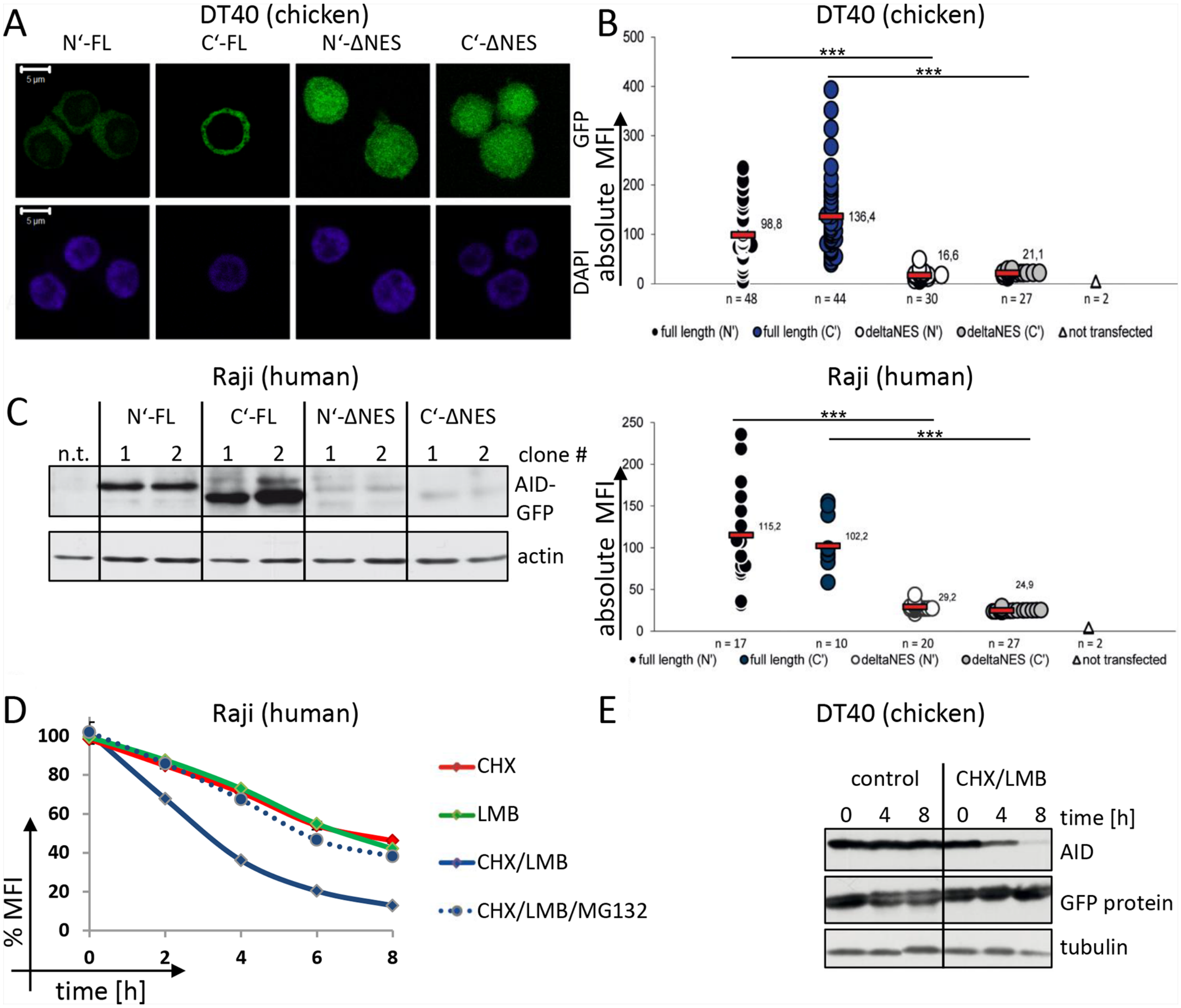


**Supplementary Figure S1: Expression and nuclear degradation of AID-GFP fusions in B cells. A.** Confocal microscopy analysis on subcellular localization of N- and C-terminally fused AID-GFP constructs in a stably transfected chicken cell line; scale bar: 5 µm. **B.** GFP fluorescence analysis by flow cytometry of the indicated AID-GFP fusions in stably transfected Raji and DT40 cells; n: number of clones analysed; red bars: mean values; significance analysis (student's *t*-test), ***: $p < 0.001$. **C.** GFP expression analysis by Western blot of two representative clones per respective construct in Raji B cells. The N- and C-terminal GFP fusions of AID run at slightly different height because the cloning inserted some more amino acids in case of the N-terminal fusion; n.t.: non-transfected cells. **D.** Nuclear degradation kinetics of AID-GFP in Raji B cells measured by FACS. **E.** Western blot analysis of nuclear degradation of AID and a GFP control protein in DT40 ΨV$^-$ cells containing an AID-IRES(*internal ribosomal entry site)*-GFP construct to express separated proteins from one mRNA transcript.

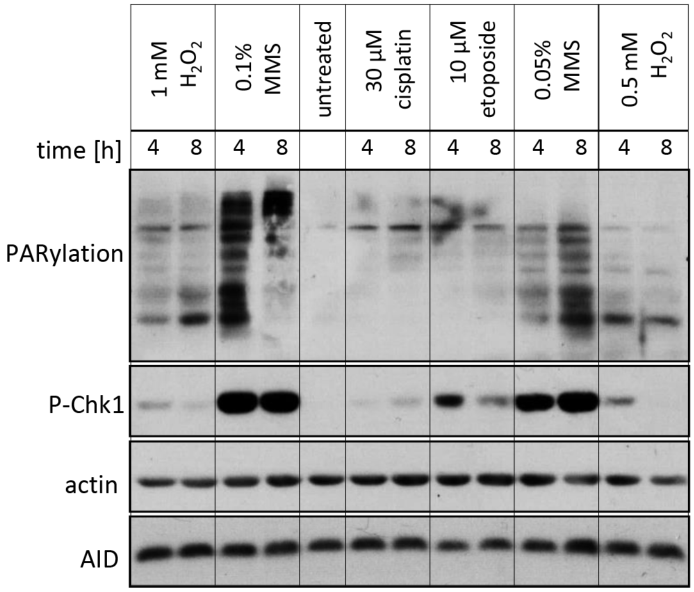


**Supplementary Figure S2: Effects of exogenous DNA damage.** Western blot analysis of the indicated proteins (AID and actin) and posttranslational modifications (Phospho-Chk1 (Ser345) and Poly(ADP-ribosyl)ation) in human Raji B cells upon treatment with different drugs.

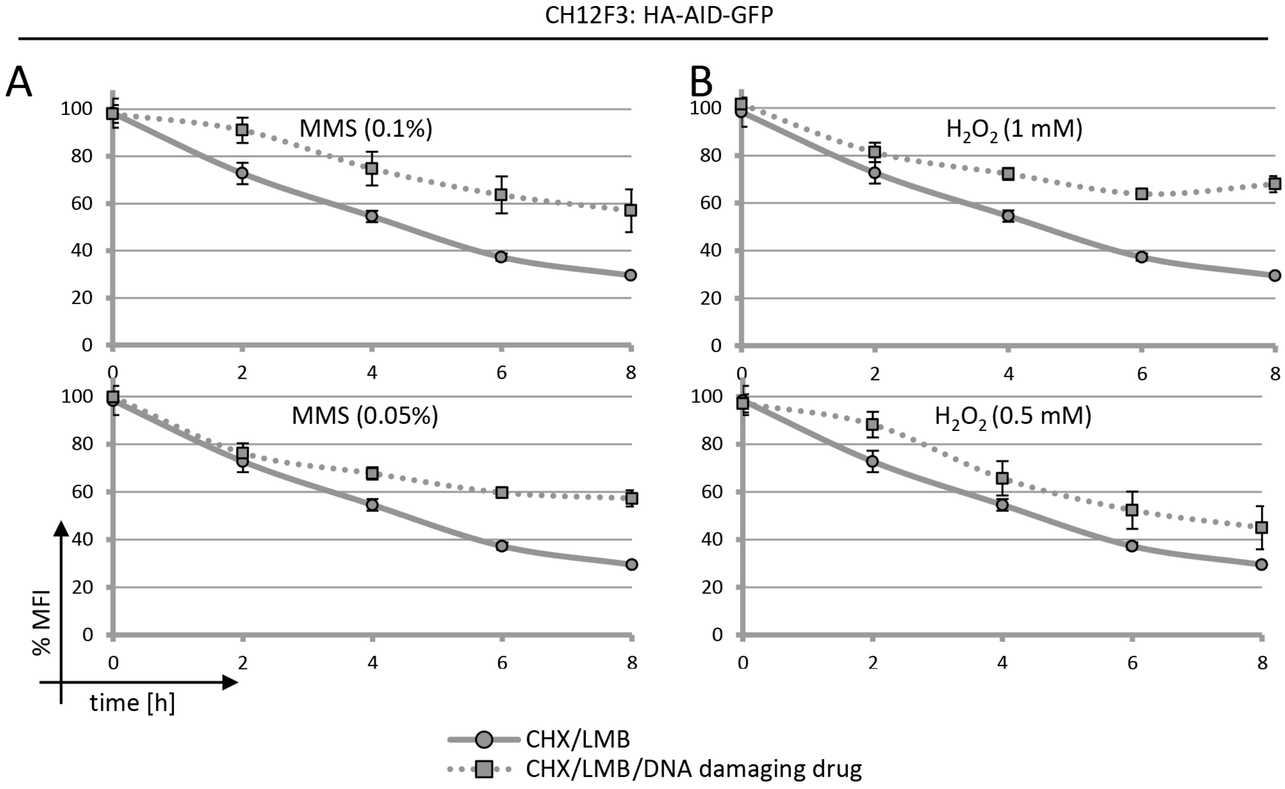


**Supplementary Figure S3: Effects of exogenous DNA damage on AID-GFP protein degradation in the mouse B cell line CH12F3. A, B.** FACS analysis of nuclear degradation of the AID-GFP proteins upon treatment with MMS or $H_2O_2$. Relative MFI values are given as a function of time with the indicated variation for three independent clones.

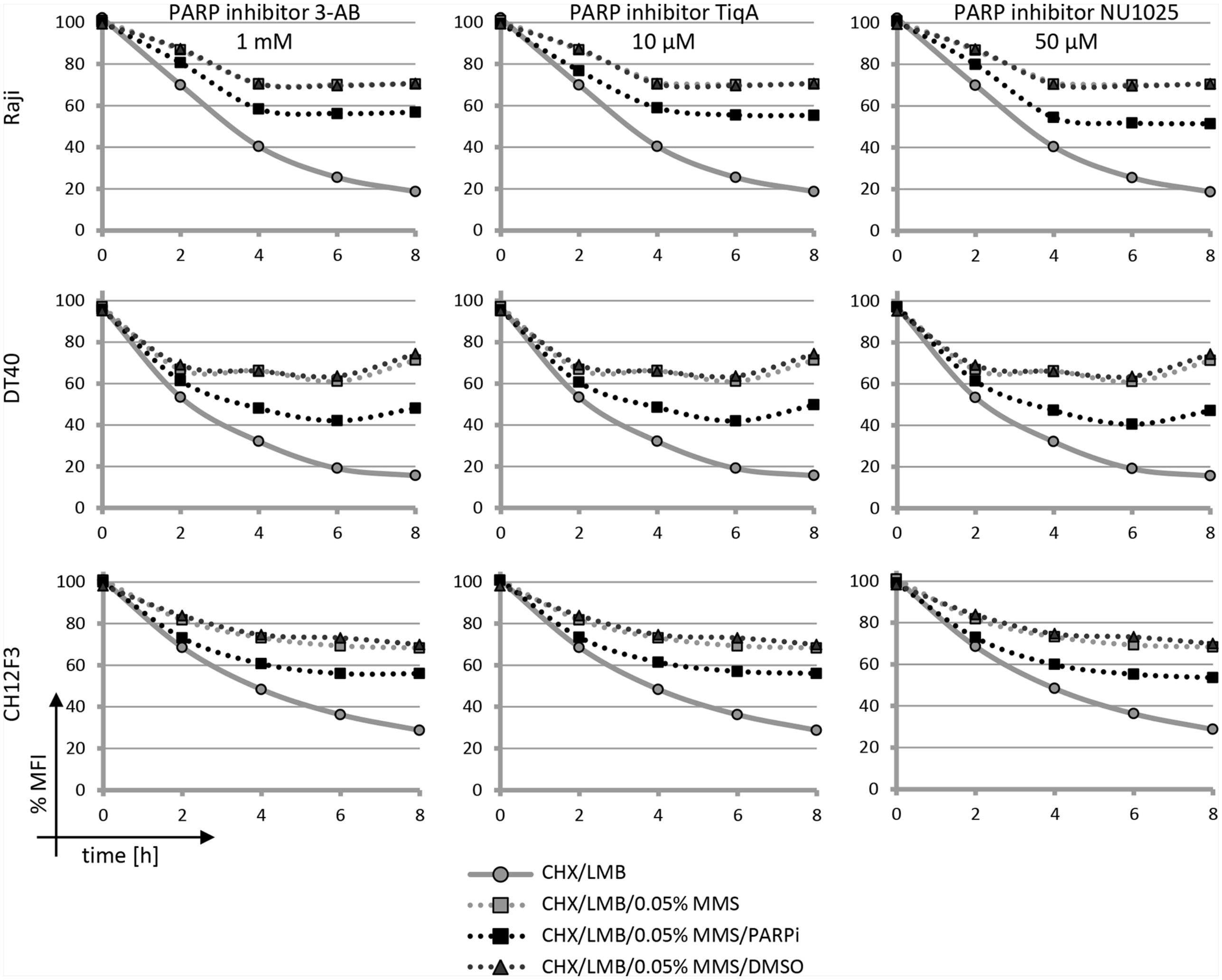


**Supplementary Figure S4: Impact of PARP inhibition on AID-GFP degradation after exogenous DNA damage for different cell lines.** FACS data of AID-GFP nuclear degradation kinetics upon treatment with 0.05% MMS and the indicated PARP inhibitor or DMSO. Relative MFI values are shown for human Raji, chicken DT40 and mouse CH12F3 B cells stably expressing an AID-GFP construct.

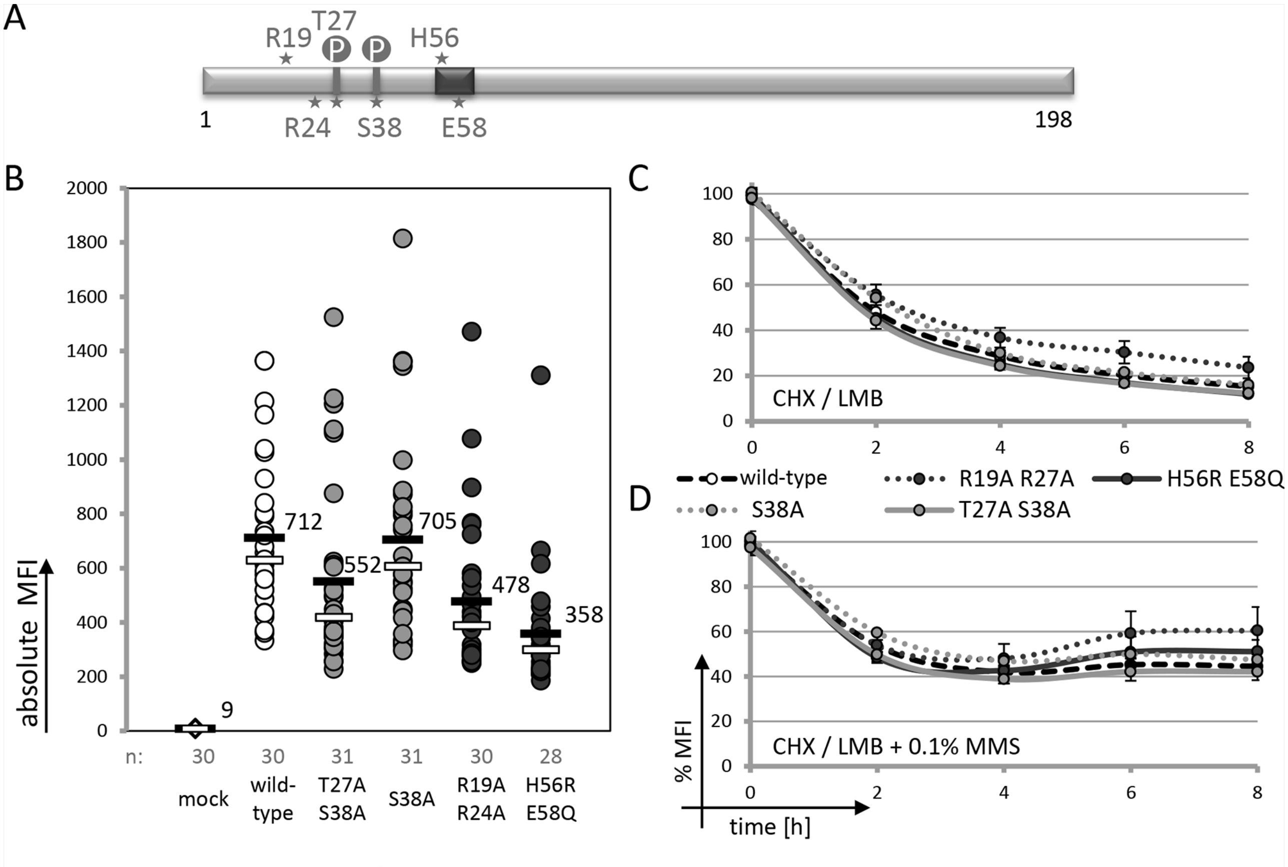


**Supplementary Figure S5: Expression and degradation of AID mutants. A.** Schematic presentation of the AID mutants designed on the AID-GFP background in the pCAGGs vector. Grey bar: AID coding sequence; black box: deaminase motif; P: phosphorylation site;*: mutation site of the respective amino acid. **B.** GFP expression analysis of the AID mutants in DT40 ΨV$^{-}$AID$^{-/-}$ B cells; n: number of clones analysed per respective mutation; black bars: mean values; white bars: median values. **C, D.** Comparative FACS analysis of nuclear degradation of AID-GFP and its respective mutants without (C) and with (D) 0.1% MMS treatment. Data were collected in the same experiment. For each clone an untreated sample is set to 100% MFI. Error bars indicate the standard deviation for five clones per construct.

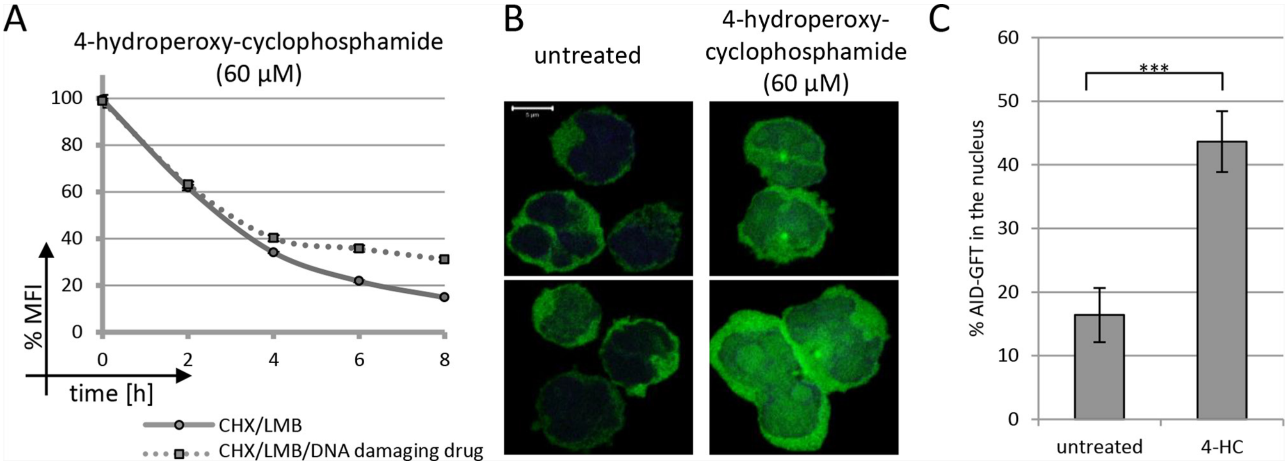


**Supplementary Figure S6: The active metabolite of cyclophosphamide stabilizes AID in the nucleus in human B cells. A.** FACS data of AID-GFP nuclear degradation kinetics upon treatment with 4-hydroperoxy-cyclophosphamide. Relative GFP MFI values are given as a function of time with the indicated standard deviation for quadruplicates in the Raji B cell line stably expressing an AID-GFP construct. **B.** Localization of AID-GFP after 6 hours of treatment with 4-hydroperoxy-cyclophosphamide (4-HC); scale bar: 5 µm. **C.** Quantification of the experiment shown in (B), analyzing 8-11 cells per condition; significance analysis (student's *t*-test), ***: $p < 0.001$

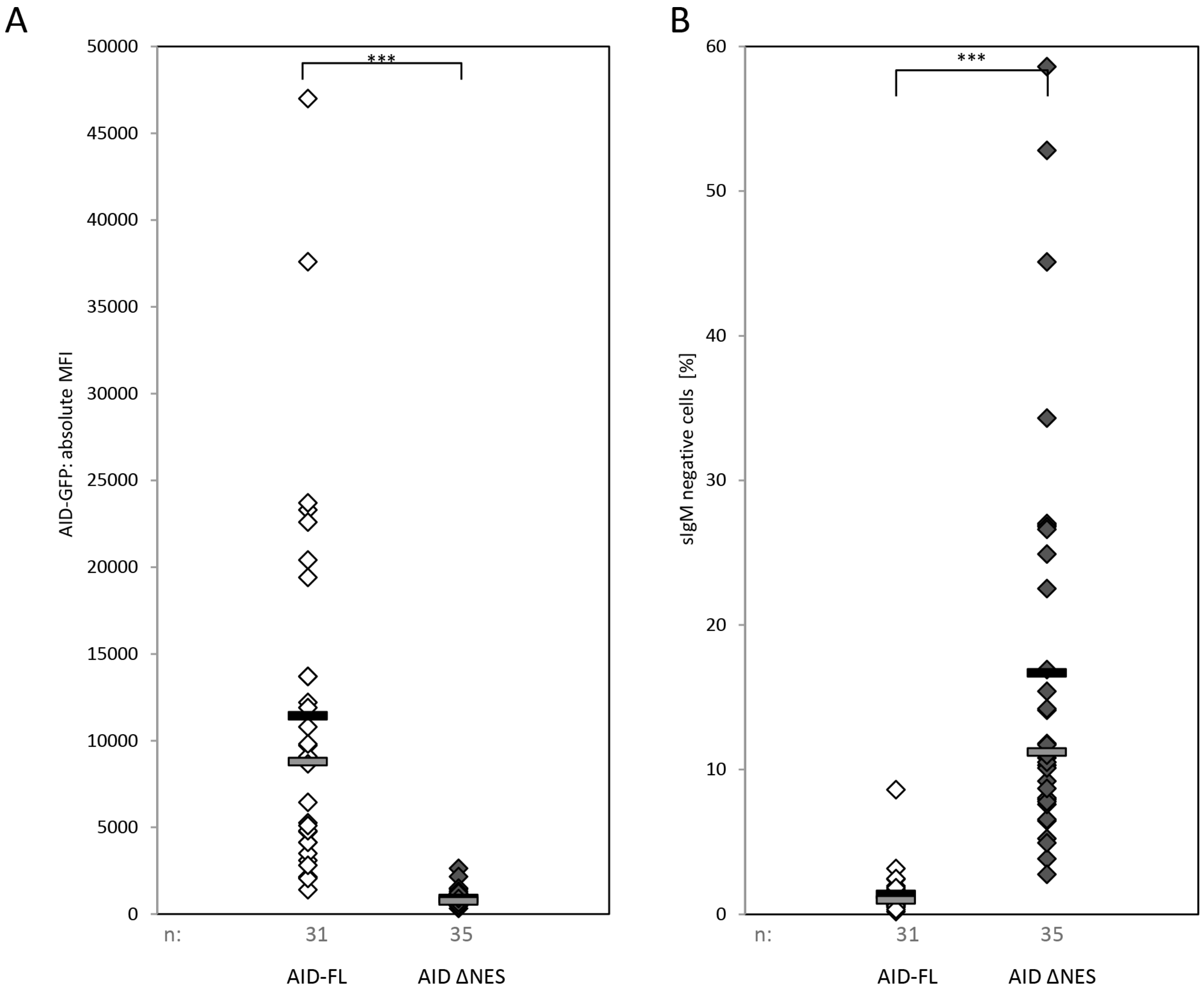


**Supplementary Figure S7: The influence of AID localization on somatic hypermutation. A.** GFP fluorescence analysis by flow cytometry of single cell clones derived by transfection of the indicated AID-GFP fusions (full length (FL) versus delta nuclear export sequence (ΔNES)) into DT40 $\Psi V^{-}$ $AID^{-/-}$. **B.** Somatic hypermutation was quantified by loss of sIgM (surface IgM) expression upon culture of the clones for 19 days. Hence, the hypermutation activity is much higher with nuclear AID despite its lower expression; n: number of single cell clones analyzed; black bars: mean values; grey bars: median values; significance analysis (student's *t*-test), ***: $p < 0.001$.

**Supplementary Table S1: Primer sequences used for site-directed AID mutagenesis by PCR**

| Mutation | primer | 5‘→3‘ sequence |
|---|---|---|
| R19E/R24E | forward | AAGGGTgaGCGTGAGACCTACCT |
| | reverse | AGCCCActcGACATTTTTGAATTGG |
| S38A | forward | ACgccGCTACATCCTTTTCACTGG |
| | reverse | CACGCCTCTTCACTACGTAGCACA |
| T27A | forward | gCCTACCTGTGCTACGTAGTGAAGA |
| | reverse | CTCACGCCGACCCTTAGCCCAG |
| H56R/E58Q | forward | GcagTTGCTCTTCCTCCGCTACAT |
| | reverse | ACtctGCAGCCGTTCTTATTGCGAA |

Lower case letters give the exchanged nucleotides. R19E/R24E and H56R/E58Q double mutants were generated in a one-step PCR procedure, the T27A mutation was generated on the basis of a S38A-mutated AID sequence in a second round of mutagenesis.
